# The Interplay of Core Diameter and Diameter Ratio on the Magnetic Properties of Bistable Glass-Coated Microwires

**DOI:** 10.3390/mi15111284

**Published:** 2024-10-22

**Authors:** Valeria Kolesnikova, Irina Baraban, Alexander Omelyanchik, Larissa Panina, Valeria Rodionova

**Affiliations:** 1 Research and Education Center “Smart Materials and Biomedical Applications”, Immanuel Kant Baltic Federal University, 236041 Kaliningrad, Russia; irinmachay@gmail.com (I.B.); aleksander.omelianchik@ext.unige.it (A.O.); drlpanina@gmail.com (L.P.); vvrodionova@kantiana.ru (V.R.); 2 Department of Materials Technology of Electronics, National University of Science and Technology «MISIS», 119049 Moscow, Russia

**Keywords:** bistability, magnetization process, glass-coated microwires, FORC-analysis

## Abstract

Glass-coated microwires exhibiting magnetic bistability have garnered significant attention as promising wireless sensing elements, primarily due to their rapid magnetization switching capabilities. These microwires consist of a metallic core with diameter *d*, encased in a glass coating, with a total diameter *D*. In this study, we investigated how the dimensions of both components and their ratio (*d*/*D*) influence the magnetization reversal behavior of Fe-based microwires. While previous studies have focused on either *d* or *d*/*D* individually, our research uniquely considered the combined effect of both parameters to provide a comprehensive understanding of their impact on magnetic properties. The metallic core diameter *d* varied from 10 to 19 µm and the *d*/*D* ratio was in the range of 0.48–0.68. To assess the magnetic properties of these microwires, including the shape of the hysteresis loop, coercivity, remanent magnetization, and the critical length of bistability, we employed vibrating sample magnetometry in conjunction with FORC-analysis. Additionally, to determine the critical length of bistability, magnetic measurements were conducted on microwires with various lengths, ranging from 1.5 cm down to 0.05 cm. Our findings reveal that coercivity is primarily dependent on the *d*/*D* parameter. These observations are effectively explained through an analysis that considers the competition between magnetostatic and magnetoelastic anisotropy energies. This comprehensive study paves the way for the tailored design of glass-coated microwires for diverse wireless sensing applications.

## 1. Introduction

Over the past few decades, magnetically soft amorphous microwires have gained renewed attention due to their remarkable properties, including magnetic bistability, higher harmonic generation, and the giant magnetoimpedance effect [1,2,3,4,5]. Magnetically bistable Fe-based microwires are of particular interest for their unique magnetization reversal processes [6,7]. In bistable microwires, the magnetization reversal occurs through the exceptionally fast domain wall movement along the wire’s axis, known as a large Barkhausen jump [7]. This process requires a critical external magnetic field (referred to as the switching field) and a specific length threshold—the critical length of bistability [8]. The dependence of the switching field on external factors such as mechanical stress and temperature enables its use in various wireless sensors [9,10,11]. Furthermore, identification tags that facilitate the tracking of goods or products can also be based on fast magnetization switching. Microwire samples that exhibit bistability when exposed to an alternating external magnetic field generate sharp voltage pulses containing higher frequency harmonics. The spectral characteristics of the voltage response provide the required information. For all these applications, a minimal length of the microwires is desirable.

The primary determinant influencing the formation of a specific domain structure in glass-coated microwires is the distribution of internal stress generated during the Taylor–Ulitovsky manufacturing process [12]. This process involves the rapid drawing from the melt inside a glass flask followed by cooling. The resulting internal stresses are composed of quenching stresses and those arising from the different thermal expansion coefficients of glass and metal, which are primarily determined by the transverse dimensions of the microwires. Additionally, there are stresses formed during the drawing process. Several other interrelated factors impact the domain structure and, consequently, the magnetization reversal behavior. These factors encompass the wire geometrical parameters, such as the metallic core diameter, *d*, and the ratio of *d* to the total diameter, *D*; magnetostriction; external stresses [13]; conventional annealing [14]; Joule heating [15], etc. The critical length of bistability also exhibits a complex reliance on many factors such as saturation magnetization and the balance between magnetoelastic and magnetostatic energies [16]. As the magnetoelastic energy is intricately linked to the distribution of internal stress, which is notably influenced by the transverse dimensions (*d* and *d*/*D*), these parameters are most important in shaping the magnetic bistability properties of these microwires.

Previous studies [17,18,19] have explored the impact of geometrical parameters on the critical lengths of bistability for both amorphous microwires and glass-coated microwires. For glass-coated microwires, these investigations primarily focused on the influence of either *d* or *d*/*D*. However, to gain a comprehensive understanding of the mechanisms governing the formation of magnetic structures leading to magnetic bistability, it is crucial to consider the impact of both, *d* and *d*/*D*. This combined influence determines the balance between different stresses and serves as the focal point of our current research. Along with hysteresis loop measurements, a first-order reversal curve (FORC) analysis was performed to elucidate the complex magnetic interactions when the wire length was nearly equal to the critical length. This was particularly useful for understanding the domain structure transformation at the critical length of the wire.

## 2. Materials and Methods

The investigated amorphous glass-coated microwires were produced by the Taylor–Ulitovskiy technique from an alloy of Fe_77.5_Si_7.5_B_15_ composition with a positive magnetostriction coefficient [13]. The diameter of the metallic core, *d*, ranged from 10 to 19 µm, and the ratio of *d* to the total microwire diameter *D* varied in the range of 0.48–0.68. The parameters of the investigated microwires are presented in Table 1.

Magnetic properties of the samples were investigated using a vibrating sample magnetometer (VSM, Lake Shore, Model 7400). For each composition, the hysteresis loops were obtained for different sample lengths in the range of 1.5–0.05 cm. A first-order reversal curve (FORC) analysis [20] was used to analyze the transformation in a domain structure at a critical length. The FORC analysis procedure is a valuable tool for revealing the magnetic interactions. It provides insights into the evolution of magnetic phases within complex systems [21,22,23]. To experimentally obtain FORCs, the sample under investigation first needed to be magnetically saturated. Subsequently, the external magnetic field was decreased to some value *H*_r_ (return field) and then the curve spanning from *H*_r_ to the saturation field was measured. This curve constitutes a single FORC. The experimental approach involved measuring numerous FORCs for each sample to cover the entire area of the hysteresis loop. Each FORC was compared in terms of switching field distributions (SFDs). The SFD represents the first derivative of magnetization with respect to the applied magnetic field (*H*_a_) [24]. For each sample, the hysteresis loops and FORCs were obtained by VSM using the in-plane geometry of the magnetic field.

## 3. Results and Discussion

**Figure 1:**
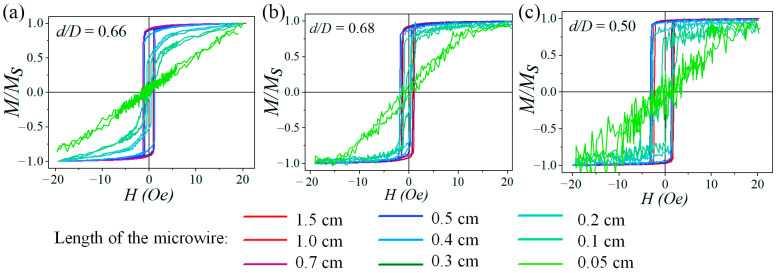
Hysteresis loops for different lengths of amorphous glass-coated microwires with Fe_77.5_Si_7.5_B_15_ composition of the metallic core with transverse parameters: (**a**) *d*/*D* = 19/29 (µm) = 0.66, (**b**) *d*/*D* = 13/19 (µm) = 0.68, (**c**) *d*/*D* = 13/26 (µm) = 0.50 (*H* is the external magnetic field, *M* is the magnetization as a function of *H*, and *M*_s_ is the saturation magnetization).

Figure 1 illustrates the hysteresis loops of amorphous glass-coated microwires with an Fe_77.5_Si_7.5_B_15_ metallic core with varying core to total diameter rations of 19/29, 13/19, and 13/26 µm. Figure 1a,b compare samples with similar *d*/*D* ratios of 0.66–0.68 but different values of *d*. The magnetization curves shown in Figure 1b,c are for samples with the same core diameter *d* = 13 µm but different *d*/*D* ratios. These hysteresis loops clearly depict the transition from initially rectangular to inclined profiles as the length of the microwire decreases. The critical length is shorter for microwires with a smaller value of *d*.

The shortening of the microwire results in what is commonly referred to as hysteresis ‘*s*moothin*g*’, accompanied by a reduction in remanent magnetization (*M*_r_). The appearance of ‘*s*moothin*g*’ regions signifies that, apart from the rapid propagation of the domain wall, the magnetization reversal process within a certain microwire volume involves magnetization rotation. We can calculate the volume of the axially magnetized core using the squareness coefficient: *K* = *M*_r_/*M*_s_. For example, for the sample with parameters *d*/*D* = 19/29 = 0.66 (Figure 1a), the volume of the axially magnetized core at 1.5 cm length is 92% (*K* = 0.92), while it reduces to 55% (*K* = 0.55) at a length of 0.2 cm. For the microwire with *d*/*D* = 13/19 = 0.68 (Figure 1b), the squareness coefficient reduces from 0.93 at the maximum studied length to 0.75 at a length of 0.2 cm. The same reduction dynamics are observed for the microwire with *d*/*D* = 13/26 = 0.50 (Figure 1c); at a sample length of 1.5 cm *K* = 0.96, which is larger than for the other studied samples, the impact of the ‘*s*moothin*g*’ regions on the hysteresis loop is smaller. This behavior stems from an increase in the volume of closure domains relative to the volume of the dominant axial domain located at the microwir*e*’s center. However, the microwire can still be considered bistable, if the ‘*s*moothin*g*’ region occupies only a small fraction of the total hysteresis loop area (typically a few percent). Further shortening of the microwire eventually leads to the loss of bistability, reaching a critical length that depends on various parameters associated with the reverse domain formation.

A microwire with *d* = 19 µm (Figure 1a) maintains its magnetic bistability up to a length of 0.3 cm. At a length of 0.2 cm, approximately 45% of the microwire volume is occupied by closure domains, where the magnetization reversal process takes place mainly by rotation. Consequently, the microwire can no longer be considered bistable, and the critical length of bistability for microwires with parameters *d*/*D* = 19/29 µm is determined to be 0.3 cm. Further shortening of the microwire to 0.1 cm results in a closure domain volume comparable to that of the axially magnetized core at the microwir*e*’s center, leading to a reduction in the coercive force. At lengths less than 0.1 cm, the magnetization curve corresponds to an orthogonal configuration between the magnetic field and the magnetization, so the microwire reverses magnetization through rotation only. The same behavior is observed for microwires with a core diameter of 13 µm (Figure 1b,c). However, the critical length of bistability for these microwires is shorter—0.1 cm. It can be inferred that as the microwire length decreases, the axial domain initially divides into two opposing magnetized regions (domains) of equal magnitude, driven by the need to maintain the energy balance and prevent stray fields.

**Figure 2:**
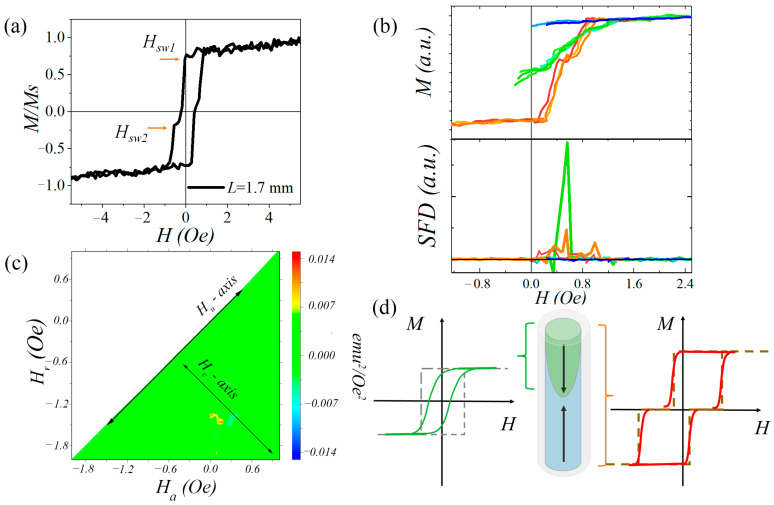
(**a**) The hysteresis loop, (**b**) set of FORCs and SFDs, (**c**) FORC diagram and (**d**) illustration of the magnetization process and the domain structure of a single Fe_77.5_Si_7.5_B_15_ (*d*/*D* = 13/26 µm) microwire at critical length (*H*_r_ is the reversal field, *H*_a_ is the applied field, and SFD is the switching field distribution).

Figure 2a illustrates the hysteresis loop of a single Fe_77.5_Si_7.5_B_15_ (*d*/*D* = 13/26 µm) microwire at a length nearly equal to the critical length, showing an additional step. The two-stepped hysteresis suggests the presence of two magnetostatically interacting hysterons, as described by the Preisach hysteresis model [25]. The Preisach model provides a basis of the mathematical description of a magnetic hysteresis process. In this model, a system of interacting magnetic entities (called hysterons) is introduced, which are completely characterized by the switching fields that are distributed. In this context, a hysteron is a mathematical operator that acts on the magnetic field and produces a square hysteresis loop. Instead of switching fields, it is convenient to use a coercive field $H_{c}$ (half-width) and an interaction field $H_{u}$ (horizontal bias). The main idea of the Preisach model is that the hysteresis loop of any material can be represented as the superposition of interacting magnetic hysterons with distributed parameters $\;H_{c}\;and\;H_{u}$ [25,26].

Previously, in ref. [26], the FORC analysis procedure was used to study the magnetization process in two magnetostatically interacting Fe-based bistable microwires. Here, we employed the same method to understand the magnetic hysteresis behavior at a critical length, using, as an example, one Fe_77.5_Si_7.5_B_15_ microwire sample (*d*/*D* = 13/26 = 0.50). Figure 2b shows the sets of FORCs for the studied microwire. Three main sets of FORCs are distinguished by different shades of color. The first set of FORCs (blue shades) corresponds to return field values smaller than the first switching field of the sample: |*H*_r_| < |*H*_sw1_|. The second set (green shade) is observed when the return field values are larger than the first switching field but smaller than the second one (corresponding to the additional plateau in the hysteresis loop): |*H*_sw1_| < |*H*_r_| < |*H*_sw2_|. The third set (red shades) represents return field values larger than the second switching field, encompassing the magnetization process of the entire system: |*H*_r_| > |*H*_sw2_|.

For each FORC set, the switching field distribution (SFD) was calculated as the first derivative of the magnetic moment with respect to the external magnetic field. For the first FORC set (blue), the SFDs are around zero. For the second set of FORCs, we observe that nearly half of the magnetic volume of the system is involved in the magnetization process. Given that the Fe-based amorphous metallic core is structurally homogeneous along its length, we conclude that this volume corresponds to a magnetic domain occupying almost half of the metallic core volume. For this domain, rather large SFDs values are seen, indicating a fast magnetization process. The green lines do not form a rectangular shape, but instead show some tilt, which is different from that predicted by the magnetic hysteron behavior. This can be explained by assuming that the magnetization process in half of the microwire (as described by the green FORCs) occurs in two regimes: the fast propagation of the domain wall and the rotation of the magnetic moment. The SFDs of the third set of FORCs show two peaks, which are attributed to the switching process of the first and second magnetic domains in the microwire. These domains switch sequentially and interact magnetostatically. The SFDs peaks (red) also show non-zero values. Similar to the green FORCs, we can infer that the magnetization process of the second magnetic domain also involves a combination of fast domain wall propagation and the magnetic moment rotation.

Figure 2c shows the FORC diagram for the microwire at the critical length characterized by two connected red-orange spots representing the irreversible switch. In a system with two magnetic hysteron-like domains coupled by a magnetostatic interaction, these spots are expected to be separated [26,27]. However, below the critical length, the closure domain structure becomes more complex and the magnetization process can involve additional domain wall jumps and reversible magnetic moment rotation. This effect is likely visible on the diagram as the additional area connecting the two main FORC peaks.

Figure 2d shows the proposed idealized scheme of the Fe-based microwire’s domain structure at the critical length along with hysteresis loops. The right pattern depicts the processes occurring in the entire system: dark brown dashed hysteresis represents the idealized behavior with two irreversible magnetization switches, while our interpretation is shown in red, which reflects the presence of rotational processes as well. The left pattern shows the magnetization change within a single domain in the same fashion: the green dashed hysteresis represents the idealized switching behavior and the realistic one is shown by a green solid loop. We assume that for the microwire with a critical length and a two-stepped hysteresis pattern the domain structure consists of two magnetic domains. The magnetization process occurs through the sequential switching of each magnetic domain, involving both fast domain wall propagation and the rotation of the magnetic moment vector.

**Figure 3:**
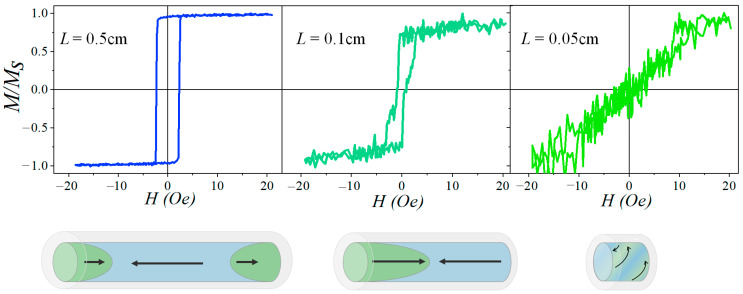
Hysteresis loops of amorphous glass-coated microwires of Fe_77.5_Si_7.5_B_15_ composition with parameters *d*/*D* = 13/26 = 0.50 for different lengths (*L*) and a schematic representation of the corresponding micromagnetic structure.

Therefore, in bistable microwires with a length nearly equal to the critical length, the axial domain divides into two domains with opposite magnetization. Figure 3 illustrates the hysteresis loop and a schematic representation of the anticipated micromagnetic structure as the sample length changes. It is evident that at a length of 0.5 cm, almost the entire volume of the microwire is occupied by the axially magnetized core, and the predominant process of magnetization reversal is the rapid propagation of the domain wall along the microwire axis. As the length decreases to 0.1 cm, which equals the critical length, the micromagnetic structure comprises two nearly equal and oppositely magnetized domains, resulting in a two-step magnetization reversal process. For a length of 0.05 cm, which is below the critical length, the magnetization reversal process consists solely of the rotation of the magnetic moment vector.

An analysis of the hysteresis loop shape indicates that the diameter of the ferromagnetic core influences the critical length of bistability. Specifically, the critical length is greater for microwires with a larger diameter of the ferromagnetic core, primarily due to the relatively larger size of the closure domains in these microwires.

**Figure 4:**
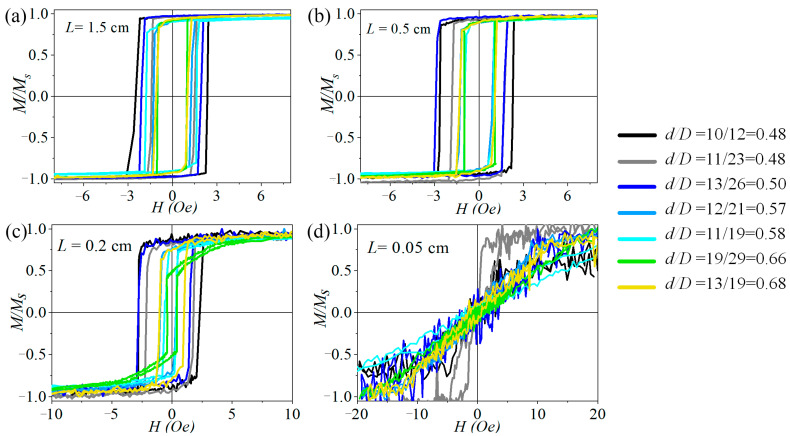
Hysteresis loops for microwires with different ratios of diameters. The length of the sample: (**a**) *L* = 1.5 cm, (**b**) *L* = 0.5 cm, (**c**) *L* = 0.2 cm, (**d**) *L* = 0.05 cm.

To explore the impact of the diameter ratio of the microwires, that is, the internal stress values, we compared the hysteresis loops of the microwires with different transverse geometric parameters but the same length. These hysteresis loops are depicted in Figure 4.

For diameter ratios ranging from 0.48 to 0.5, the hysteresis loops exhibit an ideal bistable behavior for sample lengths down to 0.2 cm, whereas the other microwires show ‘*s*moothin*g*’ regions at these lengths. In Figure 4c (*L* = 0.2 cm), the ‘*s*moothin*g*’ regions of these microwires become more pronounced, and the bistability vanishes at a length of 0.05 cm (Figure 4d). The magnetization process for all microwires, except those with a diameter ratio of *d*/*D* = 11/23, occurs through the rotation of the magnetic moment rather than the domain wall propagation. Therefore, it can be inferred that microwires with a smaller diameter ratio possess a higher level of stress, indicating a greater anisotropy energy. Consequently, these microwires maintain bistable behavior even at shorter sample lengths.

**Figure 5:**
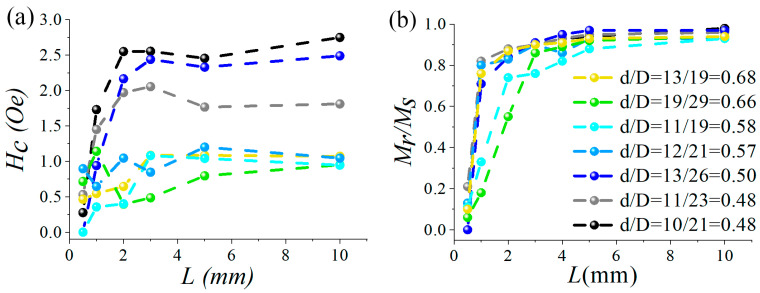
Dependence of coercive force *H*_c_ (**a**) and *M*_r_/*M*_s_ (**b**) on the wire length for different diameter ratios. The color legend is the same for (**a**,**b**).

Figure 5 depicts the relationship between coercive force (*H*_c_) and reduced remanence (*M*_r_/*M*_s_) as functions of the sample length for various values of *d* and *d*/*D*. As the bistable state is approached, both *H*_c_ and *M*_r_/*M*_s_ exhibit a sharp increase with increasing length, followed by a plateau. This behavior is associated with the evolution of the domain structure, as previously discussed. Notably, *H*_c_ is considerably higher for smaller values of *d*/*D* (in particular, for *d*/*D* ranging from 0.48 to 0.5), as one would anticipate due to the higher internal stress in these microwires. There is also a general trend of decreasing coercivity with increasing diameter ratio. However, the value of *d* alone does not have a pronounced effect on coercivity. For instance, microwires with the same diameter of 10 µm but differing values of *d*/*D* exhibit significantly different *H*_c_ values.

## 4. Conclusions

In this study, we have investigated both the individual and combined influences of the diameter of the metal core and the ratio of the metal core diameter to the total diameter of glass-coated microwires on their magnetic properties. The key findings are as follows:

(1) As the length of the microwire decreases, both the coercive force and remanent magnetization also decrease. This phenomenon results from the increasing relative volume of the closure domains, and the rise in the demagnetizing factor along the microwire’s axis. Consequently, the magnetization reversal of the microwires occurs not only due to the rapid movement of the domain walls but also due to the rotation of the magnetic moments within the closure domains.
(2) FORC analysis of the microwire at its critical length revealed magnetostatic interactions between the magnetic domains inside the microwire like those observed in a system of two bistable microwires.
(3) Our analysis demonstrated that as the *d*/*D* ratio decreases, both the coercive force and remanent magnetization increase. This effect is attributed to the higher internal mechanical stresses resulting from the different thermal expansion coefficients of the metal and glass components. These increased stresses lead to higher magnetoelastic anisotropy within the core, strengthening a preferred orientation of the magnetic moment along the microwire axis, particularly at very short lengths, down to 0.1 cm. It is noteworthy that this small critical length for magnetically bistable microwires has been identified for the first time, particularly in a microwire with a *d*/*D* ratio of 0.5.

These findings are significant for a deeper understanding of the magnetic properties of glass-coated microwires under varying transverse geometric and length parameters, paving the way for miniaturization in applications such as sensing technologies and magnetic coding systems.

## Figures and Tables

**Table 1:** Diameter of the metallic core (*d*), total diameter of the glass-coated microwire (*D*), and diameter ratio (*d*/*D*) of the investigated amorphous glass-coated microwires with Fe_77.5_Si_17.5_B_15_ composition of the metallic core.

№	*d*, µm	*D*, µm	*d*/*D*
1	10	21	0.48
2	11	23	0.48
3	13	26	0.50
4	12	21	0.57
5	11	19	0.58
6	19	29	0.66
7	13	19	0.68

## Data Availability

The original contributions presented in the study are included in the article, further inquiries can be directed to the corresponding author.

## References

[1] Nematov M.G., Kolesnikova V., Evstigneeva S.A., Alam J., Yudanov N.A., Samokhvalov A.A., Andreev N., Podgornaya S.V., Soldatov I., Schaefer R. (2021). Excellent soft magnetic properties in Co-based amorphous alloys after heat treatment at temperatures near the crystallization onset. J. Alloys Compd..

[2] Alam J., Bran C., Chiriac H., Lupu N., Óvári T.A., Panina L.V., Rodionova V., Varga R. (2020). Cylindrical micro and nanowires: Fabrication, properties and applications. J. Magn. Magn. Mater..

[3] Jiang Q., Qiao Y., Xiang C., Uddin A., Wu L., Qin F. (2022). Metacomposite based on three-dimensional ferromagnetic microwire architecture for electromagnetic response. Adv. Compos. Hybrid Mater..

[4] Al Ali M., Platko P., Bajzecerova V., Kusnir S., Kmet S., Nalevanko S., Spegarova A., Galdun L., Varga R. (2023). Application of bistable glass-coated microwire for monitoring and measuring the deformations of metal structural members. Meas. J. Int. Meas. Confed..

[5] Panina L.V., Dzhumazoda A., Nematov M.G., Alam J., Trukhanov A., Yudanov N.A., Morchenko A.T., Rodionova V., Zhukov A. (2019). Soft Magnetic Amorphous Microwires for Stress and Temperature Sensory Applications. Sensors.

[6] Calle E., Jiménez A., Vázquez M., del Real R.P. (2018). Time-resolved velocity of a domain wall in a magnetic microwire. J. Alloys Compd..

[7] Zhukova V., Corte-Leon P., González-Legarreta L., Talaat A., Blanco J.M., Ipatov M., Olivera J., Zhukov A. (2020). Review of domain wall dynamics engineering in magnetic microwires. Nanomaterials.

[8] Chichay K., Rodionova V., Zhukova V., Ipatov M., Perov N., Gorshenkov M., Andreev N., Zhukov A. (2020). Tunable domain wall dynamics in amorphous ferromagnetic microwires. J. Alloys Compd..

[9] Kozejova D., Varga R. (2023). Bistable magnetic microwire for contactless sensor of intracranial pressure. J. Magn. Magn. Mater..

[10] Churyukanova M., Kaloshkin S., Shuvaeva E., Stepashkin A., Zhdanova M., Aronin A., Aksenov O., Arakelov P., Zhukova V., Zhukov A. (2018). Non-contact method for stress monitoring based on stress dependence of magnetic properties of Fe-based microwires. J. Alloys Compd..

[11] Praslička D., Blažek J., Šmelko M., Hudák J., Čverha A., Mikita I., Varga R., Zhukov A. (2013). Possibilities of measuring stress and health monitoring in materials using contact-less sensor based on magnetic microwires. IEEE Trans. Magn..

[12] Rodionova V.V., Baraban I.A., Panina L.V., Bazlov A.I., Perov N.S. (2018). Tunable Magnetic Properties of Glass-Coated Microwires by Initial Technical Parameters. IEEE Trans. Magn..

[13] Zhukova V., Corte-Leon P., Blanco J.M., Ipatov M., Gonzalez-Legarreta L., Gonzalez A., Zhukov A. (2022). Development of Magnetically Soft Amorphous Microwires for Technological Applications. Chemosensors.

[14] Corte-Leon P., Zhukova V., Blanco J.M., Ipatov M., Taskaev S., Churyukanova M., Gonzalez J., Zhukov A. (2021). Engineering of magnetic properties and magnetoimpedance effect in Fe-rich microwires by reversible and irreversible stress-annealing anisotropy. J. Alloys Compd..

[15] Vahovsky O., Thiaville A., Varga R., Richter K. (2020). Manipulation of Domain Wall Shape in Thin Magnetic Wire by Current Annealing. Acta Phys. Pol. A.

[16] Vázquez M., Chiriac H., Zhukov A., Panina L., Uchiyama T. (2011). On the state-of-the-art in magnetic microwires and expected trends for scientific and technological studies. Phys. Status Solidi Appl. Mater. Sci..

[17] Vázquez M., Zhukov A.P. (1996). Magnetic properties of glass-coated amorphous and nanocrystalline microwires. J. Magn. Magn. Mater..

[18] Zhukov V.L.A., Vazquez M., Velazquez J., Hernando A. (1997). Magnetic properties of Fe based glass-coated microwires. J. Magn. Magn. Mater..

[19] Squire P.T., Aktkinson D., Giggs M.R.J., Atalay S. (1994). Amorphous Review Article wires and their applications. J. Magn. Magn. Mater..

[20] Béron F., Ménard D., Yelon A. (2008). First-order reversal curve diagrams of magnetic entities with mean interaction field: A physical analysis perspective. J. Appl. Phys..

[21] Breth L., Fischbacher J., Kovacs A., Oezelt H., Schrefl T., Brueckl H., Czettl C., Kührer S., Pachlhofer J., Schwarz M. (2023). FORC diagram features of Co particles due to reversal by domain nucleation. J. Magn. Magn. Mater..

[22] Martínez-García J.C., Rivas M., Lago-Cachón D., García J.A. (2015). FORC differential dissection of soft biphase magnetic ribbons. J. Alloys Compd..

[23] Kolesnikova V.G., Makarova L.A., Omelyanchik A.S., Sobolev K.V., Isaev D.A., Alekhina I.A., Komlev A.S., Rodionova V.V., Perov N.S. (2022). Magnetoactive elastomers based on ferromagnetic and ferroelectric particles: A FORC approach. J. Magn. Magn. Mater..

[24] Rivas M., Gorria P., Muñoz-Gómez C., Martińez-García J.C. (2017). Quasi-Static AC FORC Measurements for Soft Magnetic Materials and Their Differential Interpretation. IEEE Trans. Magn..

[25] Mayergoyz I.D., Friedman G. (1988). Generalized preisach model of hyst eresis (invited). IEEE Trans. Magn..

[26] Kolesnikova V., Martínez-García J.C., Rodionova V., Rivas M. (2020). Study of bistable behaviour in interacting Fe-based microwires by first order reversal curves. J. Magn. Magn. Mater..

[27] Cabanas A.M., del Real R.P., Laroze D., Vázquez M. (2023). First-Order Reversal Curves of Sets of Bistable Magnetostrictive Microwires. Materials.

